# The Local Mission: Improving Access to Surgical Care in Middle-Income Countries

**DOI:** 10.1007/s00268-020-05882-8

**Published:** 2021-01-02

**Authors:** Eric S. Nagengast, Naikhoba C. O. Munabi, Meredith Xepoleas, Allyn Auslander, William P. Magee, David Chong

**Affiliations:** 1 Division of Plastic and Reconstructive Surgery, Keck School of Medicine, University of Southern California, 1510 San Pablo St, Suite 415, Los Angeles, CA 90033 USA; 2 Division of Plastic and Maxillofacial Surgery, Children’s Hospital of Los Angeles, 4650 Sunset Blvd, Los Angeles, CA 90027 USA; 3 Operation Smile Inc., 3641 Faculty Boulevard, Virginia Beach, VA 23453 USA; 4 Department of Preventive Medicine, Keck School of Medicine of the University of Southern California, Los Angeles, CA USA; 5 Division of Plastic and Reconstructive Surgery, Shriners Hospital for Children, 909 S Fair Oaks Ave, Pasadena, CA 91105 USA; 6 Department of Plastic and Maxillofacial Surgery, Royal Children’s Hospital, Flemington Rd, Melbourne, Australia

## Abstract

**Background:**

Billions of people lack access to quality surgical care. Short-term missions are used to supplement the delivery of surgical care in regions with poor access to care. Traditionally known for using international teams, Operation Smile has transitioned to using a local mission model, where surgical service is delivered to areas of need by teams originating within that country. This study investigates the proportion and location of Operation Smile missions that use the local mission model.

**Methods:**

A retrospective review was performed of the Operation Smile mission database for fiscal years 2014 to 2019. Missions were classified into local or international missions. Countries were also classified by their income levels as well as their specialist surgical workforce (SAO) density. As no individual patient or provider data was recorded, ethics board approval was not warranted.

**Results:**

Between 2014 and 2019, Operation Smile held an average of 144.8 (range 135–154) surgical missions per year. Local missions accounted for 97 ± 5.6 (67%) of the missions. Of the 34 program countries, 26 (76%) used local missions. Of the countries that had only international missions, six (75%) were low-income countries and the average SAO density was 1.54 (range 0.19–5.88) providers per 100,000 people. Of the countries with local missions, 24 (92%) were middle-income, and the average SAO density was 30.9 (range 3.4–142.4).

**Conclusion:**

International investments may assist in the creation of local surgical teams. Once teams are established, local missions are a valuable way to provide specialized surgical care within a country’s own borders.

## Introduction

Five billion people lack access to safe, timely, and affordable surgical care [1]. The majority of those without access to surgery live in the poorest parts of our world [2]. Many of these low-and-middle-income countries (LMICs) have a density of surgeons, anesthesiologists, and obstetricians (SAO) severely below recommended minimum level of 20 per 100,000 people [1, 3]. In addition, large proportions of the population live too far from a hospital capable of providing surgery [4–6]. Billions of people cannot afford the cost of surgical care or the cost of seeking surgical care [7, 8]. The combination of these barriers to receiving care makes innovation in the delivery of surgical care necessary.

International surgical missions are one method by which surgeons and non-governmental organizations (NGOs) attempt to improve access to surgical care. First popularized by Interplast, the surgical mission originally brought providers and supplies from resource-rich countries to resource-poor countries to provide short-term surgical services [9]. Surgical missions have been used to treat a number of conditions including hernias, congenital anomalies, burns, and obstetric fistulas among others [10–13]. Operation Smile, for example, is one of the longest running surgical NGOs that originated with a traditional surgical mission model [13]. Throughout its 38 year history, Operation Smile utilized the mission model to build partnerships and invest in the surgical health system in partner hospitals and countries [14].

Though this model provided care to thousands of patients in need, early surgical missions were met with a wide range of criticisms. Termed “humanitarian colonialism,” surgical missions were criticized for poor patient follow-up, limited local engagement, low cost effectiveness, and a paternalistic approach [15–17]. Due to these concerns, many organizations adapted their traditional mission model to improve on prior flaws primarily through increased engagement with local health care providers [18–20]. Over nearly 4 decades of evolution, Operation Smile utilized “diagonal development” in which the mission model was used to provide partner countries assistance with funding, infrastructure, and education and training [14, 15]. These investments helped local practitioners improve their skills and build their own cleft lip and palate teams and strengthen their local surgical system. Now, the organization supports those teams to carry out “local missions” in their respective countries.

We hypothesize that the local mission model is most effective in countries with a SAO density near the minimum suggested amount of 20 per 100,000. The purpose of this study is to investigate the prevalence of surgical care providers in LMICs and how that relates to the implementation of Operation Smile local surgical missions. This study also evaluates the settings in which local missions are effective and compare the utilization of local missions to the usage of international missions.

## Methods

A retrospective review was performed of the Operation Smile historical mission database from fiscal years, 2014 to 2019. Operation Smile is an international not-for-profit that has been providing free cleft surgery and related care to patients since 1982. The total number of local and international surgical missions was tabulated per year. Local missions were defined as those for which greater than 50% of the medical volunteers were from the country in which the mission was taking place. International missions were those missions in which 50% or fewer of the medical volunteers were from the country in which the mission was being conducted. Program countries were classified according to mission type: local only, international only, or both local and international. Countries were also classified by their income levels as well as their SAO density as recorded by The World Bank [21, 22]. International missions were compared to local missions for length of mission as well as number of patients treated. Lastly, the volunteer data for these missions were reviewed to determine the overall percentage of medical volunteers that were from LMICs. Comparison of means for the three groups was done using one-way ANOVA. Comparison of means of two groups was done using independent Student t tests. Statistical analysis was done using Microsoft Excel (Microsoft Corp, Redmond, WA).

## Results

**Fig. 1:**
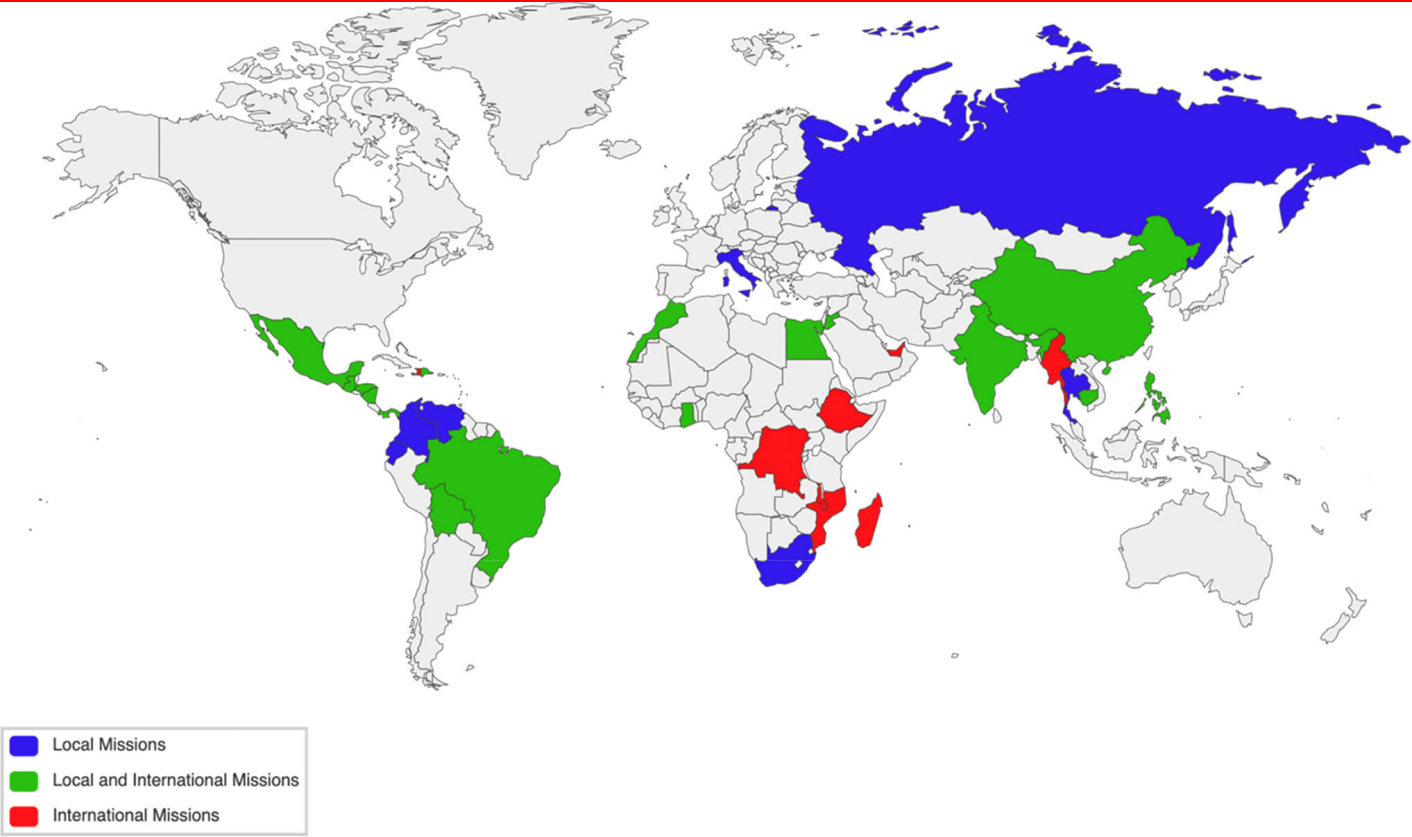
Operation Smile surgical mission countries

**Fig. 2:**
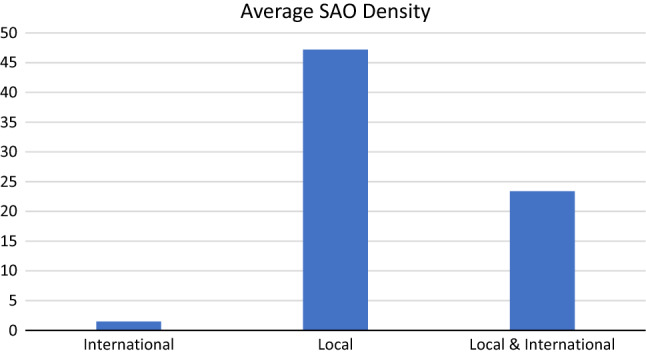
Average SAO density per country based on types of surgical missions held

Operation Smile held an average of 144.8 ± 8.6 surgical missions per year (Table 1) in 34 different countries (Fig. 1). Local missions accounted for 97 ± 5.6 (67%) of these missions. Eight countries (24%) conducted only international missions (Table 2). Of these, six (75%) were low-income countries, while one (12.5%) was a lower-middle-income country and one (12.5%) was a high-income country. The average SAO density for the countries having only international missions was 1.5 ± 2.0 providers per 100,000 people (Fig. 2). Seven (21%) countries had only local missions. Six (86%) were upper-middle-income countries, and one (14%) was a high-income country. The average SAO density of the countries with only local missions was 47.2 ± 47.2. Of the 19 countries (56%) with both types of missions, 10 (53%) were lower-middle-income, eight (42%) were upper-middle-income, and one (5%) was a high-income country. The average SAO density of these countries was 23.4 ± 17.5. The mean SAO densities between the three groups of countries are statistically significantly different (*p* = 0.01).

**Table 1:** The number of total, local, and international missions per year from 2014 to 2019

Fiscal year	Local missions, *n* (%)	International missions, *n* (%)	Total missions, *n*
2014–2015	96 (71.1)	39 (29.9)	135
2015–2016	91 (63.2)	53 (36.8)	144
2016–2017	105 (68.2)	49 (31.8)	154
2017–2018	93 (67.4)	45 (33.6)	138
2018–2019	100 (65.4)	53 (34.6)	153
Average	97.0 (67.0)	47.8 (33.0)	144.8

**Table 2:** Operation Smile surgical mission countries

Country	Classification	SAO density	Mission types
Bolivia	Lower middle income	33.87	Both
Brazil	Upper middle income	55.47	Both
Cambodia	Lower middle income	4.2	Both
China	Upper middle income	40.13	Both
Dominican Republic	Upper middle income	NA	Both
Egypt	Lower middle income	50.08	Both
Ghana	Lower middle income	NA	Both
Guatemala	Upper middle income	3.4	Both
Honduras	Lower middle income	13.68	Both
India	Lower middle income	6.82	Both
Jordan	Upper middle income	24.49	Both
Mexico	Upper middle income	NA	Both
Morocco	Lower middle income	3.66	Both
Nicaragua	Lower middle income	15.47	Both
Panama	High income	26.22	Both
Paraguay	Upper middle income	20.53	Both
Peru	Upper middle income	42.88	Both
Philippines	Lower middle income	9.56	Both
Vietnam	Lower middle income	NA	Both
DRC	Low income	0.19	International
Ethiopia	Low income	0.54	International
Haiti	Low income	5.88	International
Madagascar	Low income	0.78	International
Malawi	Low income	0.43	International
Mozambique	Low income	0.56	International
Myanmar	Lower middle income	2.42	International
UAE	High income	NA	International
Colombia	Upper middle income	22.71	Local
Ecuador	Upper middle income	59.39	Local
Italy	High income	142.4	Local
Russia	Upper middle income	63.12	Local
South Africa	Upper middle income	11.42	Local
Thailand	Upper middle income	13.09	Local
Venezuela	Upper middle income	18.13	Local

Local missions were significantly shorter (4.7 ± 0.4 days) than international missions (7.9 ± 1.1) (*p* < 0.001) (Table 3). Similarly, local missions operated on fewer patients per mission (46.1 ± 4.4) than international missions (104.1 ± 4.1) (*p* < 0.001). During these five years, the average percentage of medical volunteers who were from LMICS was 80.6% (Table 4).

**Table 3:** Number of patients treated and length of missions by mission type

	Local missions	International missions	*p* value
*n*	478	237	
Length of missions (days) (mean ± SD)	4.7 $\pm$0.41	7.9 $\pm$ 1.11	*p* = 0.0003
Patients treated per mission (mean ± SD)	46.1 $\pm$4.42	104.1 $\pm$4.10	*p* < 0.0001

**Table 4:** Percentage of medical providers from LMICs

Year	Percent of medical providers from LMICs
2015	73
2016	81
2017	81
2018	85
2019	83

## Discussion

In order to improve the inequities that exist in our world, a major focus of the World Health Organization (WHO) is health system strengthening. The WHO framework on health systems strengthening helps nations identify weaknesses in their health system and provides building blocks to achieve a strong health system [23]. One of the key take away points of the Lancet Commission on Global Surgery is that surgery should be an “integral component of a national health system in countries at all levels of development.” [1] The National Surgical Obstetric and Anesthesia Plan (NSOAP) is the framework laid out to support surgical system strengthening. After modification, the NSOAP now includes human resources, service delivery, infrastructure, financing, governance, and information management [24]. Surgical NGOs should work with ministries of health in order to work within the country’s NSOAP or health plan. Synergizing activities between players with a common goal toward health system strengthening will be crucial going forward.

Regarding human resources, the WHO has declared a critical shortage of health care providers in many parts of our world [25]. The shortage extends to all subspecialties of medicine including surgery [3]. The disparity of providers exists between countries and within countries. Most often, the poor and rural areas are most in need of surgical providers. The reality is that without providers, billions lack access to care, and many live with untreated surgical conditions [26]. Hundreds of surgical NGOs work toward improving access to surgical care, and surgical NGOs can continue to play a crucial role in the provision of surgical care while surgical systems are strengthened [1, 27, 28]. Short-term surgical missions remain a viable method to supplement surgical care for those without access to care, and they can be combined with concomitant surgical system strengthening efforts.

The gold standard for cleft care is longitudinal multidisciplinary care carried out in a cleft unit that can provide both comprehensive and complete care. Though this is the ultimate goal, it is not yet attainable in all settings. Operation Smile missions, both local and international, attempt to provide comprehensive care in a number of ways. All missions are carried out with a team of cleft surgeons, anesthesiologists, operating room nurses, recovery room nurses, surgical ward nurses, pediatricians, dentists, medical records specialists, medical photography, biomedical technicians, speech language pathologists, and child life specialists. Some missions add otolaryngologists, nutritionists, geneticists, or occupational and physical therapists [29]. Almost every partner country has a local office with local staff to help with patient coordination and team building. To help with the longitudinal aspect of care, medical records are kept on patients. All missions have a scheduled post-operative screening, and missions are carried out primarily in the same location at a similar time each year, and patient recruitment efforts exist to bring patients back for screening or further treatment.

In the poorest countries with the lowest SAO densities, Operation Smile utilizes international missions. 6 of the 8 countries where Operation Smile had only international missions are low income countries, and 5 of the 8 countries have SAO densities less than 1 per 100,000 people. No low-income countries had local missions. In these environments, subspecialty surgeons are extremely rare. The demand for cleft surgery far exceeds the capacity of the local health system [30]. Outside help is needed to provide surgical services, but surgical missions do not need to, and should not, exist without involving local health providers. Short-term, high-repetition training is an optimal environment to develop specialized surgical skills. Thus, Operation Smile created targeted training programs designed for this setting [31]. These programs are combined with education for patients, investments in infrastructure, and donations of supplies [31–33]. In fact, most Operation Smile program countries started as hosts of international missions and through diagonal development have since grown into largely self-sustained organizations.

**Fig. 3:**
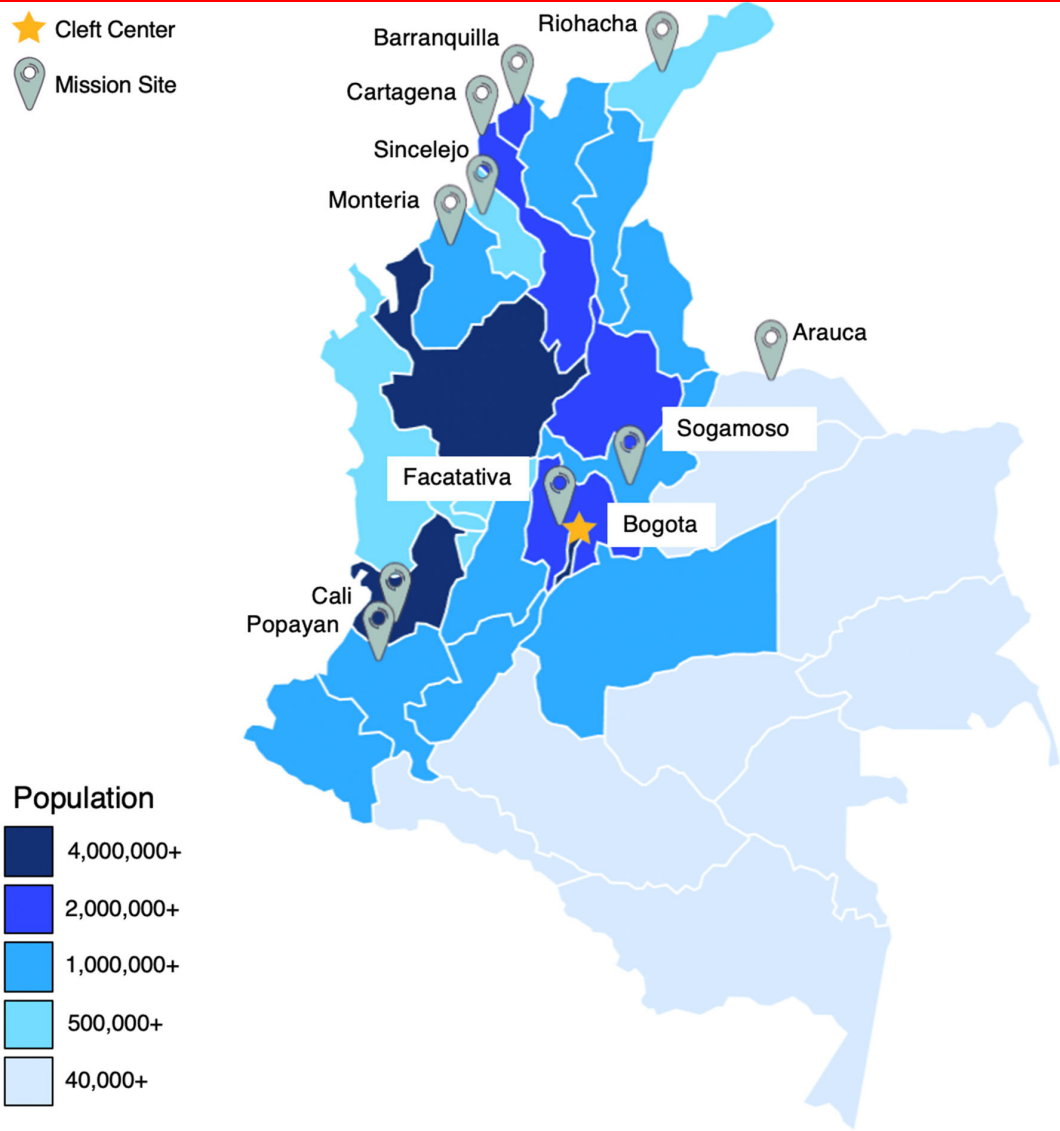
Map of Operation Smile Colombia surgical activity

Through listening to and investing in local partners, Operation Smile’ volunteer pool now consists of over 80% of medical volunteers from LMICS. Because of this volunteer distribution, 76% of Operation Smile program countries utilize local missions. These countries are typically middle-income countries with higher SAO densities than the low-income countries. Though many of these countries have SAO densities greater than the minimum recommendation of 20, middle-income countries often have regional disparities in health care providers [4–6, 34]. Local missions can take medical volunteers from urban areas to conduct cleft care in more rural settings where access to specialized surgical care remains limited. For example, Operation Smile has a center in Bogota, the largest and most densely populated city in Colombia, that runs continually and serves as the organizational hub (Fig. 3). Local missions are used to mobilize the country’s cleft surgeons to areas of need in a “hub and spokes” model.

Local missions have a number of advantages and improve upon many of the criticisms of surgical missions. For a start, local missions allow health care providers to care for patients in their own country. Patients who have complications or are too complex to receive care in the mission setting can be integrated into existing health facilities in the urban centers. Most local mission are staffed purely by local providers though some positions are scarce in certain countries and need to be supplemented with an international volunteer, most commonly speech pathologist and child life specialists. These international providers can continue to train in areas of need for the country, while the majority of care is provided by local practitioners. Local missions are shorter in duration with shorter travel time, making participation less of a burden for providers. In addition, local missions have less travel costs and less equipment shipping which has previously been shown to decrease cost per patient [35, 36].

Local missions serve to strengthen the surgical system beyond the delivery of surgical care. By bringing together local health care leaders, local missions promote camaraderie and governance. Participants work together to tackle problems in their country’s health system. Many organizations also include residents or fellows on these programs to improve their educational opportunities. Though they receive financial assistance from the international organization, local foundations fundraise for local missions providing valuable funding for surgical care in their countries where many cannot afford the cost of care. This further engages the population in advocating and improving surgical services in the country.

The ability to successfully run local missions does not necessarily make international missions obsolete, which is why so many of the countries utilize both program formats. International missions can still be utilized to help care for the existing backlog of untreated patients, especially given the greater volume of patients cared for in that setting. International missions can offer expanded educational opportunities; international experts can help with more complex cases, revisional cases, or cases not typically performed in a country. In partnership with local providers, international visitors may also contribute to identifying further opportunities for engagement. The exchange of volunteers from different backgrounds and cultures promotes teamwork and multiculturalism, which add intangible value to any organization.

This study’s main limitation is that it does not address patient outcomes between local and international mission. Previous studies have shown significant complication rates in mission settings from both international and local surgeons [37, 38]. We also do not present data on patient follow-up. This study also does not address care that takes place at Operation Smile surgical centers which play a big part in many surgical NGOs including Operation Smile. Lastly, this study is not a cost effectiveness analysis. Future investigation should focus on the economic aspects of the local mission model.

Until now, most of the discourse around supplementing surgical care has focused on international missions, mobile surgery units, or investing in surgical centers [35, 36, 39, 40]. The local mission is a concept that capitalizes on many of the benefits of investing in local surgical centers while also utilizing the flexibility of the mission model. Like other surgical missions, local missions are a concept that can be utilized for a vast array of elective surgical procedures, not just cleft lip and palate. The concept of transporting specialized surgical workforce from resource-rich to resource poor regions within a country can be used going forward by NGOS and national health care teams under the direction of the ministry of health. Local missions can act as a temporizing measure to improve access to care in middle-income countries, while the economy and the surgical health system continue to strengthen.

## Conclusion

Most of our world lacks access to quality surgical care. Surgical missions remain a valuable way to provide surgical care to those in need. International missions can be used as a means to invest in local providers, staff and infrastructure in order to build surgical capacity and strengthen the health system. Once in-country teams are created, local missions can be used as a valuable way to provide specialized surgical care within a country’s own borders. International support can still be beneficial in countries able to run local missions. This local mission model is most useful in countries where the specialized surgical workforce is strong in the urban areas, but many more rural parts of the country are without access to specialized surgical care.
